# The Economic Burden of HIV/AIDS and Myocardial Infarction Treatment in Brazil

**DOI:** 10.1155/2013/864962

**Published:** 2013-12-30

**Authors:** Denizar Vianna Araújo, Luciana Bahia, Steffan Frosi Stella

**Affiliations:** ^1^Department of Internal Medicine, State University of Rio de Janeiro, Boulevard 28 de Setembro, No. 77, Room 329, 20551-030 Rio de Janeiro, RJ, Brazil; ^2^National Institute of Science and Technology for Health Technology Assessment (IATS), CNPq, Rua Ramiro Barcelos, No. 2359, 90035-903 Porto Alegre, RS, Brazil

## Abstract

*Objective*. To analyze the expenses of HIV/AIDS and acute myocardial infarction (AMI) treatment in Brazil. *Methods*. A search in the Brazilian epidemiological database (DATASUS) on AMI and AIDS hospitalizations and their costs was done from January 1998 to December 2011. The number of HIV/AIDS cases and antiretroviral treatment (ART) costs was obtained from public Brazilian databases. *Results*. In 5 years, HIV/AIDS cases increased 38.5%, mainly in patients aged 25–49. There were 180,640 patients in ART in 2007 at a cost of R$ 3,920 per patient/year. The hospitalizations due to AIDS were stable over the last 13 years; however, the hospitalizations due to AMI have increased 78%. In 2007, the expenses with hospitalizations for HIV/AIDS and AMI (25–49 years) were approximately 0.12 and 1.52% of the Ministry of Health budget allocated to reimburse inpatient costs. The expenses on ART totaled 1.5% of the total budget (all age groups). *Conclusion*. The prevalence of HIV/AIDS is still increasing in Brazil. There are scientific evidences suggesting an increased incidence of AIM in this population. Considering the high costs for the treatment of both diseases, an economic analysis is important to alert health managers to strengthen the preventive measures to guarantee the financial sustainability of such treatment.

## 1. Introduction

The Brazilian HIV/AIDS Program was established in May 1985 [1]. In 1996, the Brazilian Unified Health System (SUS) ensured the coverage of antiretroviral drugs (ART) to all those living with HIV/AIDS in Brazil, resulting in AIDS mortality reduction [2].

Following the development of drug resistance in HIV patients, the Brazilian Ministry of Health had to provide new drugs at higher costs, investing up to R$ 551 million in antiretroviral treatment in 2003. There was an increase in the investment in subsequent years due to the acquisition of patented drugs (up to R$ 960 million in 2006). The introduction of the first fusion inhibitor, enfuvirtide, in the therapeutic regimen of multidrug-resistant patients had a strong impact on total costs. It was provided to 1,030 patients at a daily cost of US$ 22.19, the accounting for 4.4% of antiretroviral expenditure in 2006 [3].

It remains controversial whether the exposure to combination antiretroviral (ART) treatment increases the risk of acute myocardial infarction (AMI). Friis-Møller et al. conducted a prospective observational study with 23,468 patients with HIV from 11 previously established cohorts to evaluate the risk factors and the incidence of myocardial infarction. The authors concluded that ART therapy was independently associated with a 26-percent relative increase in the rate of myocardial infarction per year of exposure during the first four to six years of use [4]. The Data Collection on Adverse Events of Anti-HIV Drugs (D:A:D) study assessed the risk of AMI in 13 anti-HIV drugs in an observational study. Over 178,835 person-years, 580 patients developed AMI. Of the drugs considered, only indinavir, lopinavir ritonavir, didanosine, and abacavir were associated with a significantly increased risk of AMI (12 to 13%) [5]. Islam et al. estimated the relative risk of cardiovascular disease (CVD) among people living with HIV (PLHIV) compared with the HIV-uninfected population. The authors conducted a systematic review and meta-analysis of studies and showed that the relative risk of CVD was 1.61 (95% CI 1.43–1.81) among PLHIV without ART compared with HIV-uninfected people. The relative risk of CVD was 2.00 (95%CI 1.70–2.37) among PLHIV on ART compared with HIV-uninfected people and 1.52 (95% CI 1.35–1.70) compared with treatment-naïve PLHIV [6].

Given the importance and the high costs of treating both conditions in Brazil, this analysis aimed to estimate and analyze the costs of HIV/AIDS and AMI treatment and their impact on the public budget of the Brazilian Unified Health System.

## 2. Methods

A search in the Brazilian epidemiological database (DATASUS) on AMI and AIDS hospitalizations and their costs was done from January 1998 to December 2011. The survey was restricted to patients aged 25–49 (group with the highest prevalence of HIV/AIDS in the Brazil). Data were registered as the number of hospitalizations and reimbursed expenditures in Reais (R$). The annual number of HIV/AIDS reported cases was obtained from the TABNET database of the Brazilian Ministry of Health [7].

Data from a previously published study about the antiretroviral treatment costs were analyzed. The authors analyzed the Ministry of Health's total spending on drugs and its programs between 2002 and 2007 [8]. The Federal Government antiretroviral drug acquisitions were analyzed separately, obtained from a federal purchase website (ComprasNet) [9].

The Brazilian Public Health System perspective, which considers only direct costs that are reimbursed to health care providers was adopted. In this scenario, indirect costs are not computed in the database.

## 3. Results

**Figure 1:**
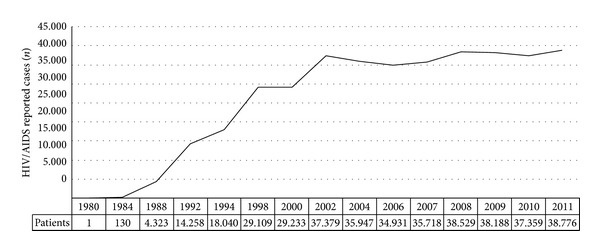
Annual HIV/AIDS reported cases in Brazil, 1980–2011.

From 1980 to June 2007, 474,273 HIV/AIDS cases were reported. In June 2012, this number increased to 656,701 registered cases (Figure 1). Data on the number of patients on antiretroviral therapy between 2002 and 2007 and the annual total and per patient costs of HIV/AIDS treatment are described in Table 1, comprising almost 100% HIV of the reported cases (Brazilian Ministry of Health, 2008).

**Figure 2:**
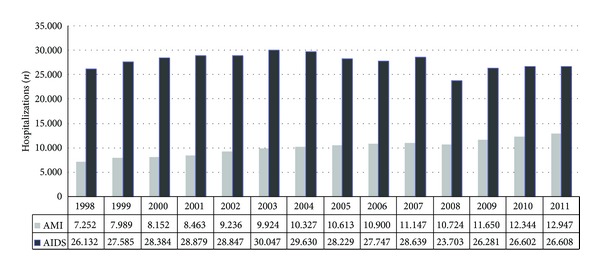
Annual hospitalizations due to AMI and AIDS (25–49 years).

**Figure 3:**
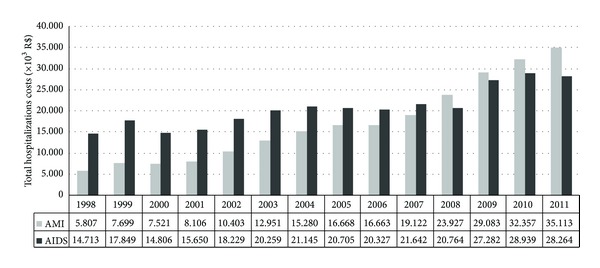
Annual costs with AMI and AIDS hospitalizations (25–49 years).

The number of hospitalizations for AMI and AIDS and their costs (patients aged 25–49; 1998–2011) is shown in Figures 2 and 3, respectively.

## 4. Discussion

In Brazil, the age-standardized mortality from cardiovascular diseases decreased by 26% in the last decade, and this decrease was partially due to the successful implementation of health policies that led to the reduction of smoking and the expansion of access to basic health care [10]. However, the prevalence of diabetes, arterial hypertension, and HIV/AIDS is increasing parallel with the prevalence of overweight and aging population, which may be reflected in unfavorable trends in mortality from cardiovascular diseases in the next decade.

Cardiovascular diseases represented the third leading cause of hospitalizations in the Brazilian Unified Health System, having been responsible for the greater spending on hospitalization, accounting for a total of R$ 1.9 billion or 19% of the total cost of hospitalizations. Acute coronary syndrome (unstable angina and acute myocardial infarction) accounted for 7% of all deaths in Brazil, associated with substantial direct and indirect costs to healthcare systems and society [11].

In the last 5 years, the number of HIV/AIDS registered cases increased 38.5% [12]. The age group where AIDS is more prevalent in both sexes is the one with 25–49 year olds, reflecting an early age group with an increased risk of developing cardiovascular events. The indirect costs, such as productivity loss in this age group, are a major concern to health policy makers and the whole society.

The number of hospitalizations due to AIDS related conditions was stable over the last 13 years; however, the number of hospitalizations due to AMI in patients aged 25 to 49 has increased approximately 78% in the same period. This age group has a low risk of cardiovascular events, but the combination of HIV exposure and HIV/AIDS treatment could be a possible reason for this increase in the incidence of AIM.

The total costs with hospitalization have increased in both conditions, but with a more substantial growth in the AMI hospitalizations costs (92% versus 504% increase for AIDS and AIM, resp.). This cost increase can be due to changes in clinical practice, such as the use of thrombolytic agents and revascularization procedures during admissions.

The total budget of the Brazilian Ministry of Health in 2007 was R$ 44.3 billion and of this total, R$ 23 billion were allocated to reimburse outpatient and inpatient treatment costs. In the same year, the expenses with hospitalizations for HIV/AIDS-related conditions and AIM (25–49 years) were approximately 0.12 and 1.52% of this budget, respectively. Its worth mentioning that, in the same year, spending on antiretroviral treatment totaled 1.5% of the total budget (all age groups).

There are 18 antiretroviral drugs and 37 formulations available in Brazil for the treatment of people living with HIV (for children and adults). Ten of these drugs are produced by multinational companies and eight are locally produced (one private and six state-run Brazilian laboratories). It has been noted that for most of these medications (79%, 11 of 14 antiretroviral drugs) there was a significant reduction in the prices between 2006 and 2007, reaching 50% reduction in some cases [8]. An analysis of the expenditures with the Brazilian antiretroviral drug program suggests that the universal access policy will not be sustainable in long term without compromising investments in other areas [13].

This study has some limitations. Firstly it adds only the indirect evidence that the rising cost of hospitalization for AMI among the low coronary risk may be due to HIV infection and the use of antiretroviral agents. Secondly, it was not possible to compare the costs of AMI in both HIV positive and negative populations, mainly due to the lack of information available in the Brazilian databases. Further work is needed to clarify this important and relevant issue.

## 5. Conclusion

The prevalence of HIV/AID is still increasing among young adults and all these individuals will have access to long-term antiretroviral treatment in Brazil. There are scientific evidences suggesting an increased incidence of myocardial infarction in this population. Considering the high costs for the treatment of both diseases, health managers and decision-makers should be aware of strengthening preventive measures in order to guarantee the financial sustainability of the treatment in public health units in Brazil.

## Figures and Tables

**Table 1:** Number of patients covered in the HIV/AIDS Program (antiretroviral drugs) and their related costs in the Brazilian Unified Health System (SUS).

Year	Number of patients	Cost (reais)	Cost per patient (reais)
2002	125,175	R$668,783,673	R$5,342
2003	139,868	R$495,900,996	R$3,545
2004	156,670	R$596,016,153	R$3,804
2005	164,547	R$567,045,691	R$3,446
2006	174,270	R$1,002,732,562	R$5,753
2007	180,640	R$708,178,407	R$3,920

Source: Vieira 2009 [8], monthly report for Assessment and Use of HIV Drugs, and STD/AIDS Program Care and Treatment Unit.
